# Secretory galectin-3 induced by glucocorticoid stress triggers stemness exhaustion of hepatic progenitor cells

**DOI:** 10.1074/jbc.RA120.012974

**Published:** 2021-01-13

**Authors:** Fan Yang, Fan Zhang, Xueying Ji, Xin Jiang, Mengjuan Xue, Huiyuan Yu, Xiaona Hu, Zhijun Bao

**Affiliations:** 1 Department of Geriatric Medicine, Huadong Hospital, Shanghai Medical College, Fudan University, Shanghai, China; 2 Shanghai Key Laboratory of Clinical Geriatric Medicine, Shanghai, China; 3 Research Center on Aging and Medicine, Fudan University, Shanghai, China

**Keywords:** Cell senescence, galectin-3, protein interaction, stemness exhaustion, liver injury, cellular senescence, galectin, stem cells, glycoprotein, protein-protein interaction, cell cycle, proliferation, AMP-activated kinase (AMPK), quiescence

## Abstract

Adult progenitor cell populations typically exist in a quiescent state within a controlled niche environment. However, various stresses or forms of damage can disrupt this state, which often leads to dysfunction and aging. We built a glucocorticoid (GC)-induced liver damage model of mice, found that GC stress induced liver damage, leading to consequences for progenitor cells expansion. However, the mechanisms by which niche factors cause progenitor cells proliferation are largely unknown. We demonstrate that, within the liver progenitor cells niche, Galectin-3 (Gal-3) is responsible for driving a subset of progenitor cells to break quiescence. We show that GC stress causes aging of the niche, which induces the up-regulation of Gal-3. The increased Gal-3 population increasingly interacts with the progenitor cell marker CD133, which triggers focal adhesion kinase (FAK)/AMP-activated kinase (AMPK) signaling. This results in the loss of quiescence and leads to the eventual stemness exhaustion of progenitor cells. Conversely, blocking Gal-3 with the inhibitor TD139 prevents the loss of stemness and improves liver function. These experiments identify a stress-dependent change in progenitor cell niche that directly influence liver progenitor cell quiescence and function.

Glucocorticoid (GC) hormones regulate essential physiological functions including energy homeostasis, embryonic and postembryonic development, and the stress response (1, 2, 3, 4). Stressful events contribute to a host of disease states if dysregulated, even affects the efficacy of anticancer therapies (5, 6, 7, 8, 9). Among important factors influencing tissue damage and aging, glucocorticoids signaling acts as a driver of stress-related damage. Psychosocial stress, circadian rhythm disorder, anxiety disorders, and abnormal metabolism always cause stress response changes including glucocorticoids pathway (10, 11, 12, 13, 14). Studies further suggest that stress-related phenotypes may together confer disease by increasing tissue aging risk (15), but the underlying mechanisms are poorly understood.

Mechanistically, the effects of stress on cellular aging could be driven by stress-responsive molecules and a reprogrammed microenvironment able to modulate function. GC stress induces epigenetic up-regulation, which contributes to aging, as well as induces protein degradation that cause muscle atrophy and weakness (2, 4, 16). Studies also address the evidence supporting inflammation, which acts as part of the senescence-associated secretory phenotype (SASP), as a crucial mechanism that underlines the consequence of stress exposure (17). The niche is a conversed regulator of progenitor cell quiescence states, which represent stemness. Stress- or damage-induced secretory phenotypes influence the niche, and therefore mediate progenitor cells quiescence and function. Age-associated changes in the niche have been shown to cause a decline in stem cell number and function (18). However, the mechanisms driving stress-induced changes of niche and progenitor cells remain unknown.

Here, we address these questions in a dexamethasone (Dex)-induced GC stress mice model. We examined the Dex-induced SASP and report that Galectin-3 (Gal-3), a kind of β-galactoside–binding lectin, elevated in Dex induced mice liver, is an aged niche factor, activates progenitor cells, and consequently breaking its quiescence. Mechanistically, Gal-3 acts as an extracellular ligand of stem cell marker CD133. Furthermore, Gal-3/CD133 interaction triggers focal adhesion kinase (FAK) phosphorylation and reduced AMPK phosphorylation. We also demonstrate long-term activation of progenitor cells breaks their quiescence through regulating the cell cycle. Importantly, knockdown of p16 promotes CD133^+^ progenitor cells proliferation and stemness loss. Additionally, the Gal-3 inhibitor TD139 reduces the stemness loss and recovers liver function. Taken together, we show that Gal-3 derived from the Dex-induced aged niche causes liver progenitor cells quiescence broken and long-term stemness exhaustion. Together these findings provide molecular insights into mechanisms linking stress and SASP to progenitor cell state and liver function, thereby pointing to biomarker and intervention possibilities.

## Results

### GC stress causes liver damage and progenitor cells expansion

Cortisol is a hormone produced in the adrenal gland released to help the body deal with stress. Cortisol levels in the body are associated with stress-including circadian rhythm disorder and anxiety disorders (11, 12). These stress-associated cortisol level changes can in turn contribute to tissue damage risk and regeneration capacity by affecting the expression of molecules regulated by the damage-induced phenotypes and the state of progenitor cells in niche, thus altering cell and tissue function. Therefore, to gain insight into the mechanisms through which the stress contributes to liver function, it is relevant to examine the effects of stress on niche and progenitor cells.

**Figure 1:**
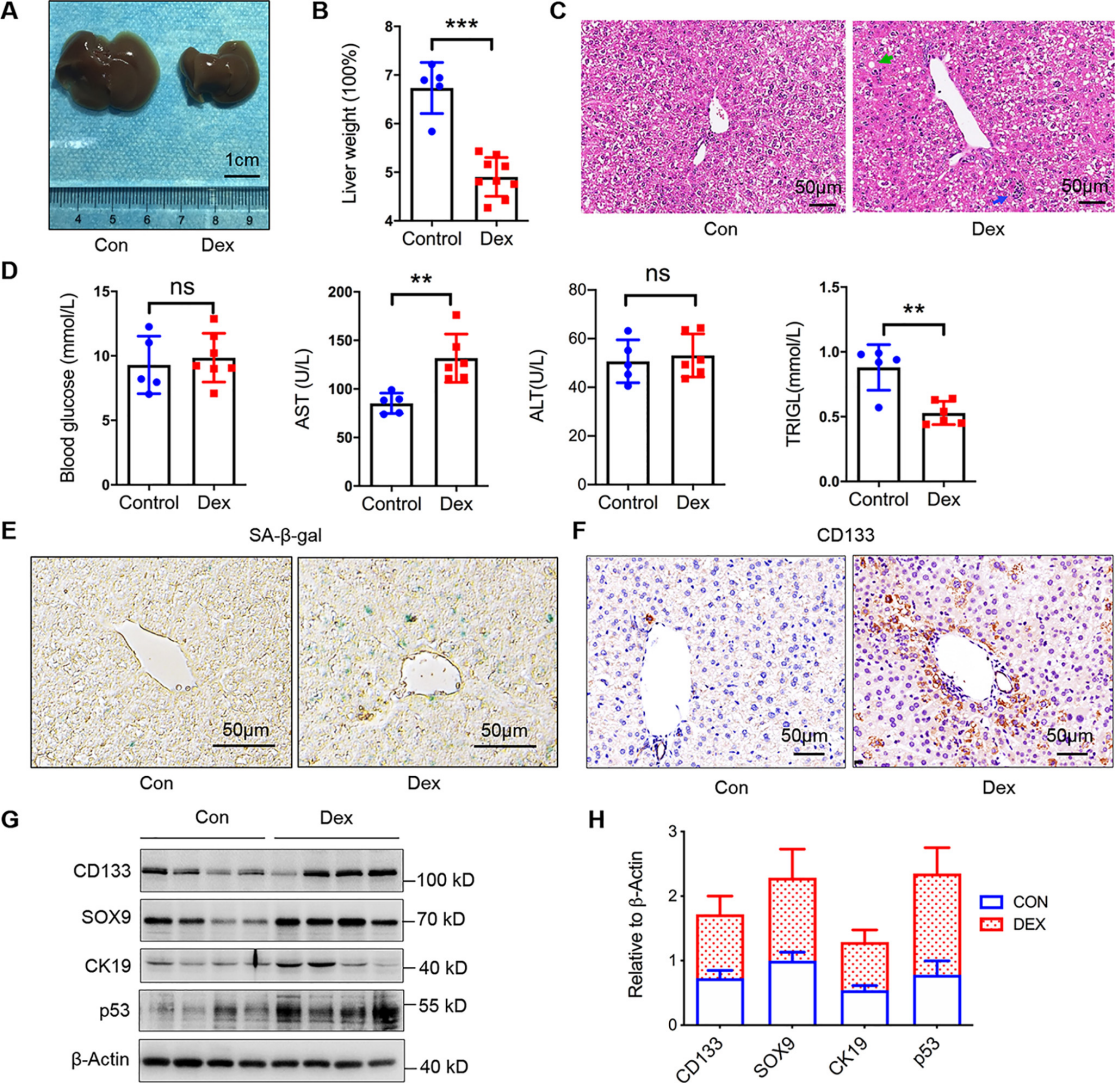
**GC stress induces cell senescent and progenitor cells expansion.***A*, representative images of mice liver. A *scale bar* is shown. *B,* the percentage of liver weight relative to body weight. Data were presented as mean ± S.D., *n* ≥ 5; ***, *p* < 0.001. *C*, histopathological examination of C57BL/6J livers. Representative images of H&E staining are shown (*scale bar*, 50 μm). Slight steatosis (*green arrow*) and punctate necrosis (*blue arrow*) are indicated. *D,* blood contents of AST, ALT, glucose, and TRIGL. Data were presented as mean ± S.D., *n* ≥ 5; **, *p* < 0.01; *ns*, no significance. *E,* liver sections were subjected to staining for β-gal activity (stained *blue*-*green*), *scale bar*, 50 μm. *F*, immunohistochemistry of CD133 for liver sections, respectively (*n* = 3). *G,* protein levels of CD133, SOX9, CK19, and p53 in mice livers (*n* = 4). β-Actin was used as a loading control. *H, grayscale* statistics of protein levels in *G*.

To identify such effects on liver, we used Dex to simulate stress in mice. We treated adult mice (8 weeks old) for 10 days with a daily intraperitoneal injection of Dex at a dose of 25 mg/kg per day. Liver weight and biochemical text were used to evaluate the liver mass and function (Fig. 1, *A, B, and D*). Hematoxylin and eosin (H&E) staining of hepatic tissues showed Dex-induced stressed liver (Fig. 1*C*). These experiments indicated that Dex caused liver damage, as indicated as slight steatosis (*green arrow*), and punctate necrosis (*blue arrow*). To examine whether the stress-associated damage induced the cellular senescence, we performed senescence β-gal staining and found that accumulated senescent cells presented in Dex-induced damaged liver (Fig. 1*E*). This result was also addressed by immunohistochemistry (IHC) detecting using SMP30 antibody, which also partially presented cellular senescence (19), less SMP30-positive cells indicated more senescent cells in the Dex group (Fig. S1A). Therefore, Dex induced senescence and changed the niche. CD133, a stem cell marker (20, 21), was used to observe the state of the liver progenitor cell. There were many CD133 positive cells presented, indicated by progenitor cells activation and expansion induced by Dex (Fig. 1*F*). Other makers of hepatic progenitor cells, such as sox9 and CK19 (22), also addressed this phenomenon and p53 was used to monitor the senescent or damaged state (Fig. 1, *G* and *H*). Together, Dex could induce cellular senescence and hepatic progenitor cell expansion.

### Quiescent state is essential for stemness maintenance

**Figure 2:**
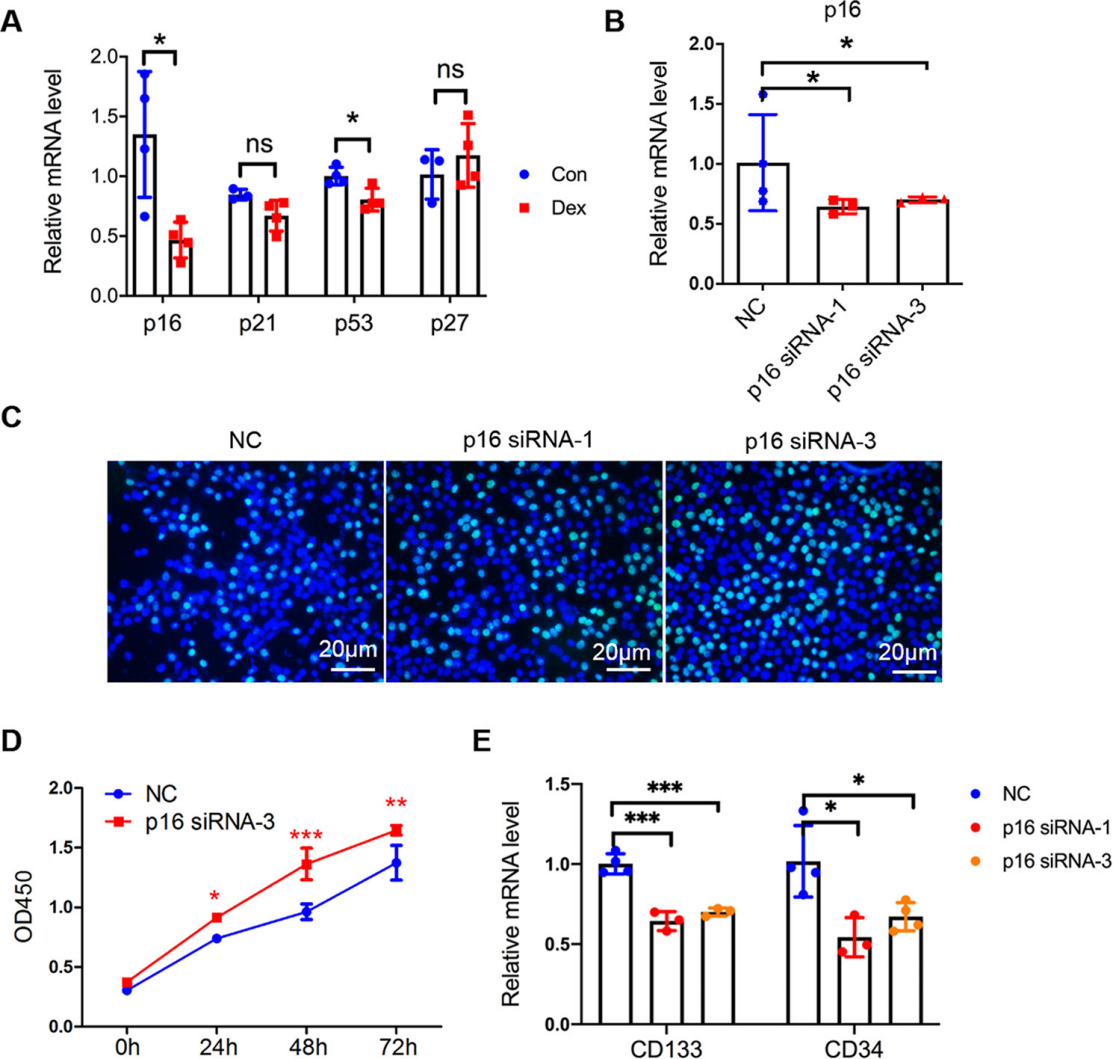
**Keeping quiescence maintains the stemness of progenitor cells.***A*, real-time qPCR analysis of p16, p53, p21, and p27 expression for mice liver. Data were presented as mean ± S.D., *n* ≥ 3; *, *p* < 0.05; *ns*, no significance. *B,* CD133^+^ huh7 cells were transfected by p16- or negative control (*NC*)-siRNA for 48 h, then expression of p16 was tested by qPCR. Data were presented as mean ± S.D., *n* ≥ 3; *, *p* < 0.05, *versus* NC group. *C*, EdU staining of CD133^+^ huh7 cells transfected by p16 siRNA for 48 h. Representative pictures of EdU^+^ (*green* stain) cells are shown. *D,* cell counting assay of CD133^+^ huh7 cells transfected by p16-siRNA or NC-siRNA. Data were presented as mean ± S.D., *n* ≥ 6; *, *p* < 0.05; **, *p* < 0.01; and ***, *p* < 0.001, *versus* NC group. *E,* qPCR analysis of CD133 and CD34 expression for CD133^+^ huh7 cells transfected by p16 siRNA for 48 h. Data were presented as mean ± S.D., *n* ≥ 3; *, *p* < 0.05; ***, *p* < 0.001, *versus* NC group.

Progenitor cell quiescence lies on one end of a spectrum of self-renewal potential spanning from quiescence, to robust proliferation, to senescence and death. Maintaining stem cell quiescence is essential for preserving the long-term self-renewal potential of the stem cell pool in the number of organ systems (23, 24). Expansion of stem cells has been implicated to induce senescence and loss of stem cell properties (18). We then hypothesized that Dex-induced activation of progenitor cells disrupted quiescence and stemness maintenance. It has reported cell cycle checkpoint genes, such as p16, p21, p53, and p27, promote cell cycle arrest, are molecular mediators of quiescence in stem cells (25, 26). To test this, we compared the mRNA levels of such genes between the Con- and DEX-treated liver tissues. The result showed that decreased p16 expression was induced by Dex (Fig. 2*A*). To further examine the role of p16 in regulating the expression of stemness markers, and stem cell proliferation, we knocked down p16 in CD133^+^ Huh7 cells. Knockdown of p16 showed an increase in cell proliferation rate (Fig. 2, *B–D*). In contrast, the expression of stem cell markers was prevented (Fig. 2*E*). These data suggest reduced p16 expression and increased proliferation cause stemness loss.

### The effect of DEX-induced high Gal-3 expression on progenitor cells activation

**Figure 3:**
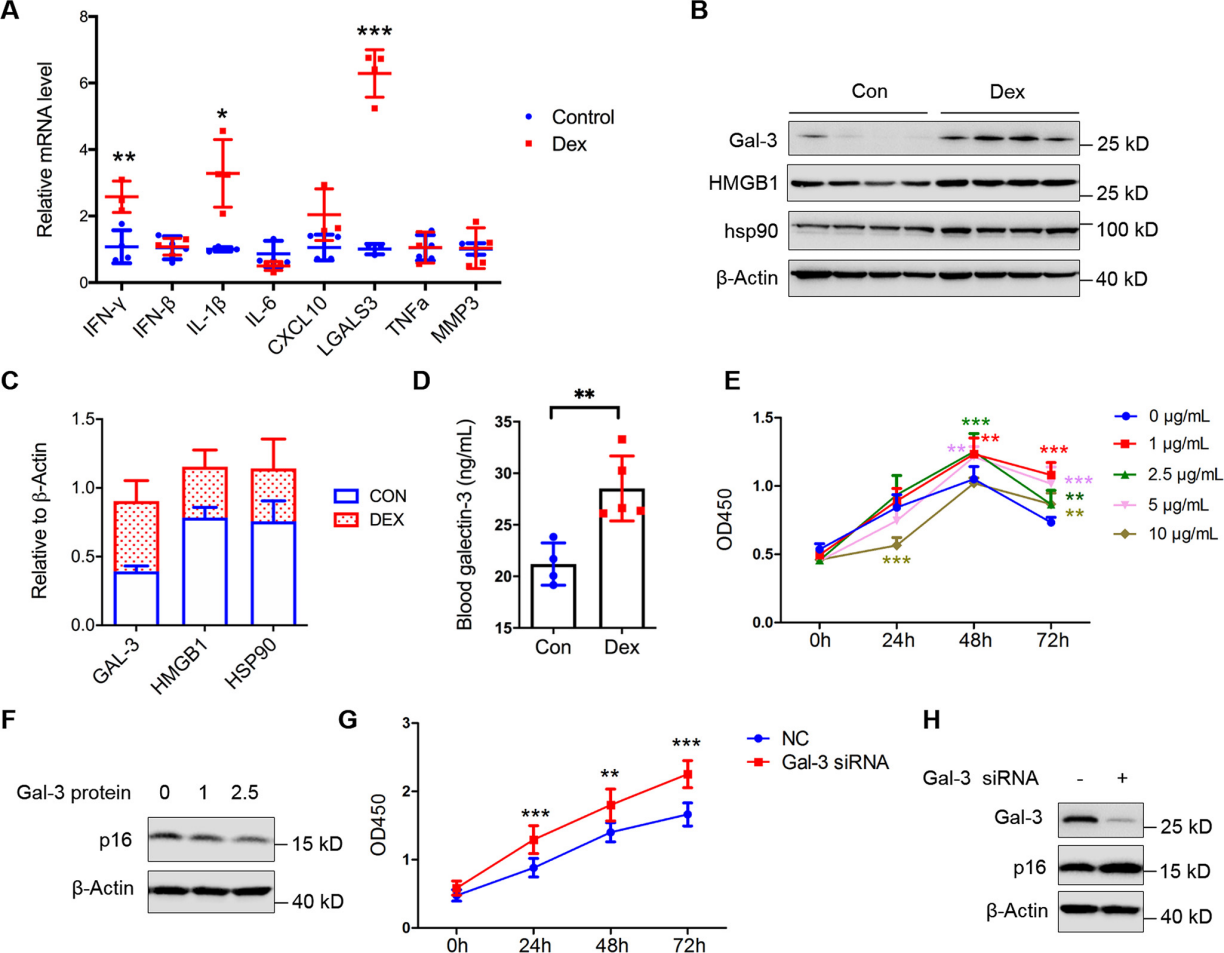
**Extracellular Gal-3 signals prevent progenitor cells in a quiescent state.***A,* qPCR analysis of SASP expression of mice liver. Data were presented as mean ± S.D., *n* ≥ 4; *, *p* < 0.05; **, *p* < 0.01; ***, *p* < 0.001, *versus* control group. *B*, Western blotting analysis of Gal-3, HMGB1, hsp90 for mice liver (*n* = 4). β-Actin was used as a loading control. *C, grayscale* statistics of protein levels in *B*. *D*, Gal-3 concentration of mice serum was measured by ELISA. *E,* CD133^+^ huh7 cells treated with indicated concentration of Gal-3 protein were subjected to perform a cell counting assay. Data were presented as mean ± S.D., *n* ≥ 8; **, *p* < 0.01; ***, *p* < 0.001, *versus* 0 μg/ml group. *F,* Western blotting detection of CD133^+^ huh7 cells cultured with the indicated Gal-3 protein μg/ml) treatments for 48 h. *G,* cell counting assay of CD133^+^ huh7 cells transfected by Gal-3-siRNA or NC-siRNA. Data were presented as mean ± S.D., *n* ≥ 6; **, *p* < 0.01; ***, *p* < 0.001, *versus* NC group. *H*, Western blotting detection of CD133^+^ huh7 cells transfected by Gal-3-siRNA or NC-siRNA for 48 h.

It is widely thought that exposure to niche factors underlines the quiescence of progenitor cells during aging. Therefore, to gain insights into the mechanisms through which factor contributes to the changeable states of progenitor cells, we asked whether disrupted quiescence was due to changes in the aged liver progenitor cell niche, it is relevant to assess the effects of SASP on progenitor cell proliferation. To identify aged niche factors that signal to hepatic progenitor cells, reverse transcription quantitative PCR was performed. Compared with the control group, LGALS3, gene name Gal-3, was remarkably increased in the DEX group (Fig. 3*A*). Western blotting detection also indicated the increased protein level of Gal-3, as well as other senescence-associated proteins (Fig. 3, *B* and *C*). The Gal-3 level in blood detected by ELISA showed that secretory Gal-3 was increased (Fig. 3*D*). Then, to determine whether Gal-3 was responsible for activating the progenitor cells, different concentrations of Gal-3 protein were added in culture medium of CD133^+^ progenitor cells to observe the effect of Gal-3 on progenitor cells proliferation. The cell counting assay showed that 1 μg/ml of Gal-3 could cause progenitor cells proliferation to be stable (Fig. 3*E*) and the external source Gal-3 could reduce p16 expression (Fig. 3*F*). We also wondered whether progenitor cell-expressed Gal-3 have the same effect. Surprisingly, knockdown of Gal-3 by siRNA showed the increased progenitor cells proliferation and high expression of p16 (Fig. 3, *G* and *H*), indicating the different effects and mechanisms of external and internal Gal-3 on progenitor cells activation. Taken together, these results suggest Gal-3 derived from the DEX-induced niche contributes to hepatic progenitor cells activation and proliferation.

**Figure 4:**
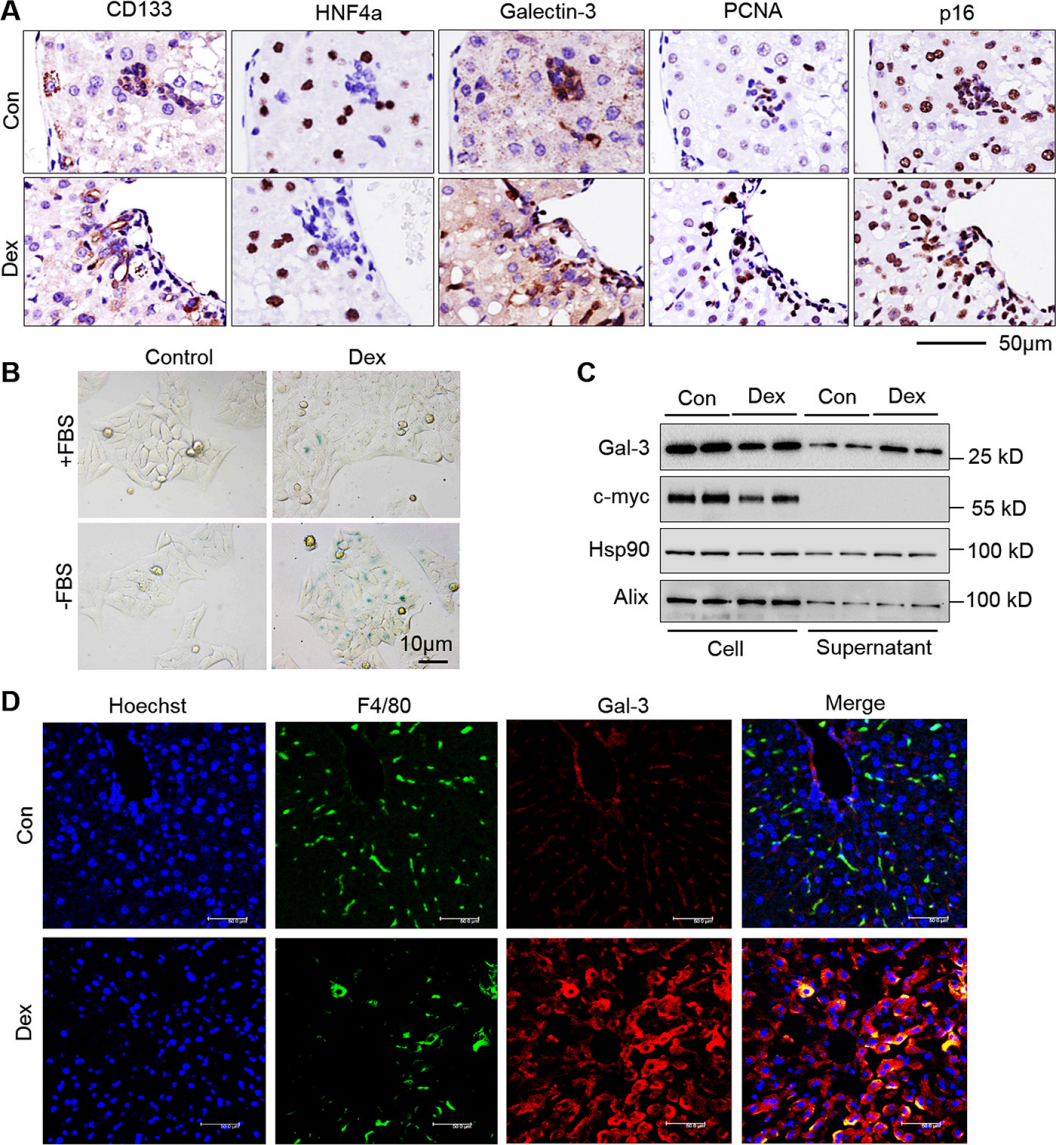
**Extracellular Gal-3 is derived from many cell types.***A,* immunohistochemistry of CD133, HNF4a, Galectin-3, PCNA, and p16 for continuous liver tissue sections, respectively. *Scale bar*, 50 μm. *B,* representative images of β-gal staining of LO2 cells treated with 50 μm Dex cultured in DMEM with or without 10% FBS for 48 h. *C*, Gal-3 expression in culture media supernatant of LO2 cells with 50 μm Dex treatment in a serum-starved condition for 48 h by Western blotting analysis. c-myc and Alix were used as intracellular and both of extracellular and intracellular control, respectively. *D*, immunofluorescent staining of F4/80 (*green*) and Gal-3 for liver tissue sections. Representative images are shown. Hoechst staining was used to visualize the nuclei of the cells. *Scale bar*, 50 μm.

To assess from which Gal-3 was derived, IHC detections using Galectin-3, CD133 (progenitor cell marker), HNF4a (hepatocyte marker), proliferating cell nuclear antigen (PCNA) (indicated proliferated cells), and p16 antibodies were performed. As indicated in Fig. 4*A*, DEX-induced and increased the number of proliferating cells; high expression of Gal-3 was derived from progenitor cells and niche; niche-derived cells expressing Gal-3 was increased. To confirm that hepatocyte is a source of niche-derived Gal-3, DEX-induced senescence in LO2 hepatocytes was performed. Cells became senescent as evidenced by the expression of senescent-associated β-gal (Fig. 4*B*). Secretory Gal-3 has also been collected from DEX- or Con-induced cell medium. Western blotting showed increased Gal-3 secretion induced by DEX and decreased intracellular Gal-3 (Fig. 4*C*). Thus, DEX could cause hepatocytes senescence and Gal-3 secretion. It is widely known that Gal-3 is also a marker of macrophages, dual-immunofluorescence staining showed that Gal-3 and F4/80 (macrophage) were strongly co-localized, indicating Gal-3 might also be derived from macrophages (Fig. 4*D*).

### Gal-3 acts as a ligand of stem cell maker CD133

**Figure 5:**
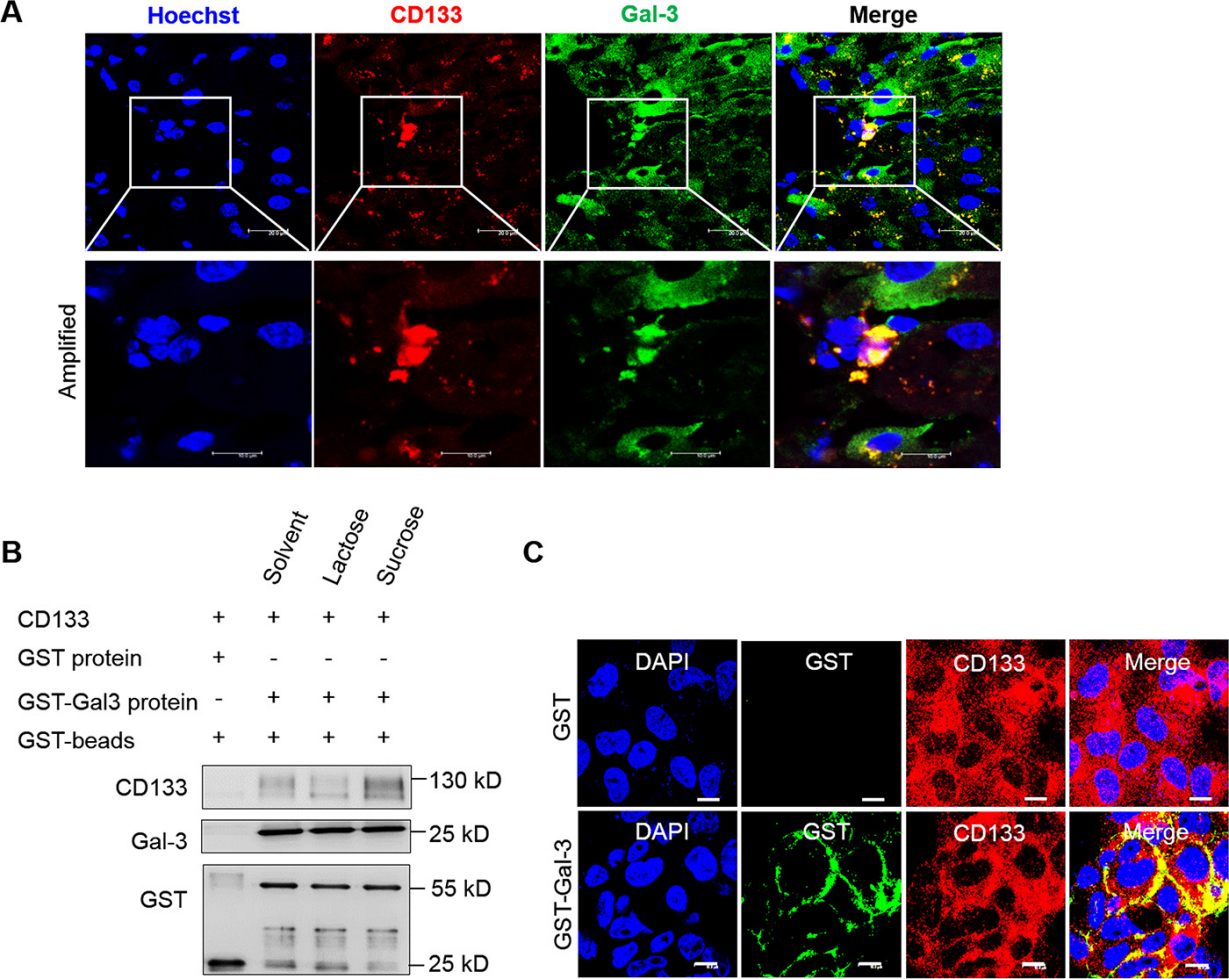
**Gal-3 interacts with stem cell marker CD133.***A*, dual-immunofluorescent staining of CD133 (*red*) and Gal-3 (*green*) on mice livers. *B*, GST pulldown assay was performed using recombinant GST-Gal-3 protein and CD133 protein purified from HEK293T cells. 0.1 m lactose or 0.1 m sucrose were added to the system for competitive inhibition. *C,* dual-immunofluorescent staining of CD133 (*red*) and GST-Gal-3 (*green*) on CD133^+^ huh7 cells. *Scale bar*, 10 μm.

We next asked whether external source-derived Gal-3 could bind to progenitor cells through a ligand-receptor interaction mechanism. CD133, a 120-kDa pentaspan transmembrane glycoprotein, has been used as a cell-surface marker of normal stem cells and cancer stem cells from a broad range of tissue types (27, 28). Our previous study has reported that CD133 contains β1,6-GlcNAc N-glycans (29), which support the possibility for Gal-3 binding (30). To confirm this, we observed co-localization of CD133 and Gal-3 in Dex-induced liver tissue. Gal-3 positive cells were localized at neighboring CD133 positive cells. CD133 and Gal-3 also co-localized in some CD133^+^ cells (Fig. 5*A*). To determine whether Gal-3 could bind to CD133 directly, we purified the full-length CD133 protein from HEK293T cells, then used Gal-3 containing GST tag to perform a GST pulldown assay. CD133 could be pulled down, indicating that Gal-3 bonds to CD133. This binding was prevented by adding lactose in a GST pulldown assay (31), which suggests interaction between Gal-3 and CD133 is in a glycosylation-dependent manner (Fig. 5*B*). Co-focus observation in CD133^+^ Huh7 cells treated by BSA or Gal-3 protein also addressed the result of the binding of Gal-3 and CD133 (Fig. 5*C*). Therefore, Gal-3 is a ligand of stem cell marker CD133.

### Gal-3 activates progenitor cells through CD133/AMPK/FAK signaling

**Figure 6:**
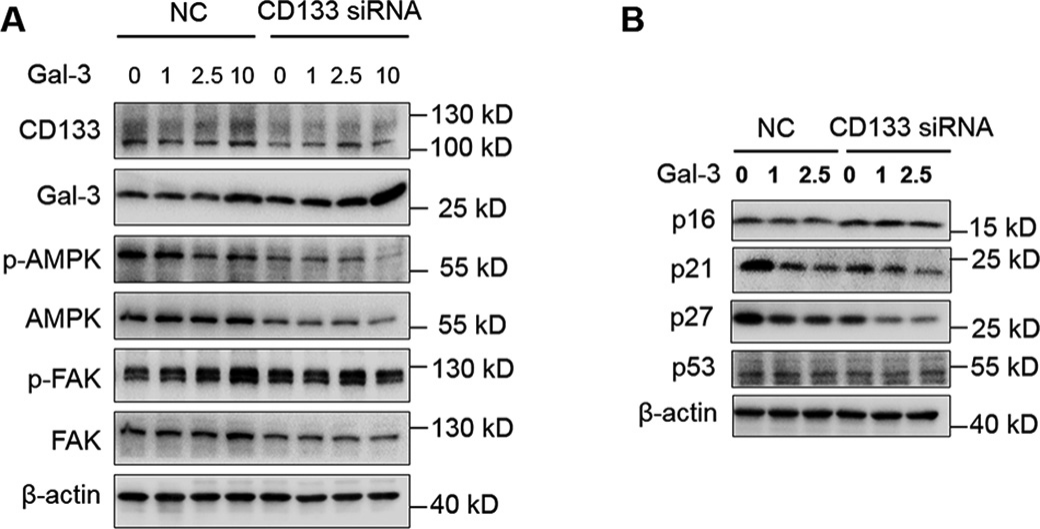
**Gal-3/CD133 interaction triggers AMPK/FAK pathway.** ACD133^+^ huh7 cells transfected with NC- or CD133-siRNA with the indicated Gal-3 protein treatment for 48 h. The expressions of Gal-3, CD133, pFAK-Tyr^925^, FAK, phospho-AMPKα (Thr^172^), and AMPKα were detected by Western blotting. β-Actin was used as a loading control. BCD133^+^ huh7 cells were transfected with NC- or CD133-siRNA with the indicated Gal-3 protein treatments for 96 h. The expressions of p16, p21, p27, and p53 were detected by Western blotting. β-Actin was used as a loading control.

AMPK is a serine/threonine protein kinase, which serves as an energy sensor in all eukaryotic cell types (32). Published studies indicate that AMPK activation strongly suppresses cell proliferation (33). To test whether Gal-3 activated the CD133^+^ cells through the AMPK pathway, we knocked down the CD133 by siRNA, then the cells were cultured in medium containing different concentrations of Gal-3 protein for 48 h. Signal was detected by Western blotting (Fig. 6*A*). FAK phos-phorylation was tested as it has been reported AMPK regulates FAK signaling in skeletal muscle. Gal-3 caused prevention of AMPK phosphorylation and increasing FAK phosphorylation in our experiments. We hypothesized that loss of quiescence driven though aged niche-derived Gal-3 signaling was contributing to cyclin-dependent kinase inhibitor depletion and long-term stemness loss. To this end, we performed a long-term Gal-3 stimulation experiment in CD133^+^ stem cells. Gal-3 protein was added in NC or knockdown of CD133 group cells cultured in medium. Western blotting detection indicated that Gal-3 induced decreased expression of p16, p21, and p27 (Fig. 6*B*). These results suggest that interaction of Gal-3 and CD133 contributes to progenitor cells activation and loss of quiescence through the AMPK/FAK pathway.

### Gal-3 inhibitor TD139 improves the aged niche-induced stemness loss and liver dysfunction

**Figure 7:**
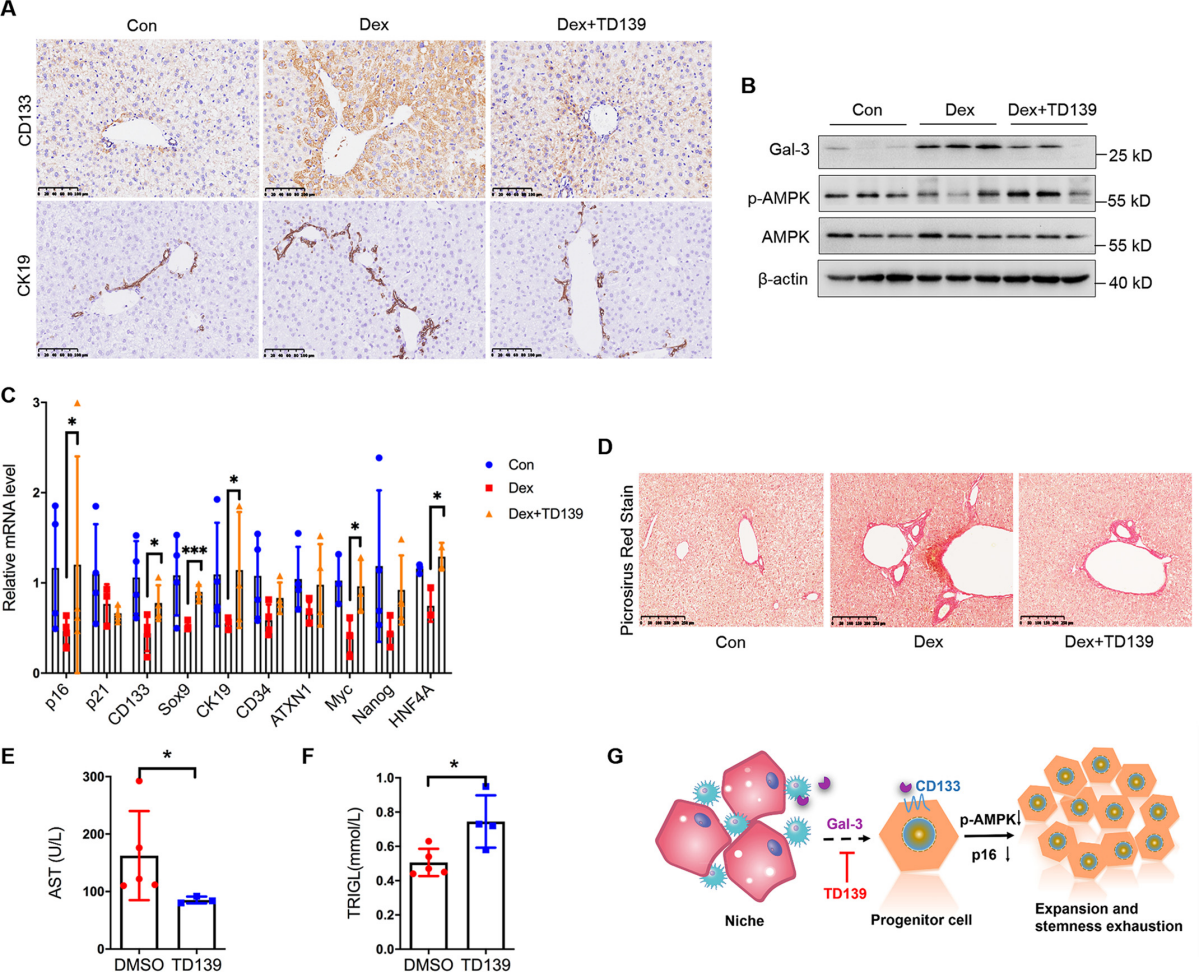
**TD139 blocks Gal-3 and restores liver stemness and function.***A*, IHC analysis of CD133 for mice liver (*n* = 3), *scale bar* is shown as indicated. *B*, Western blotting detection of Gal-3, phospho-AMPKα (Thr^172^) and AMPKα of mice liver. β-Actin was used as a loading control. *C,* real-time qPCR analysis of p16, p21, CD133, and other stemness genes expression for mice liver. Data were presented as mean ± S.D., *n* ≥ 3; *, *p* < 0.05; ***, *p* < 0.001, *versus* Dex group. *D,* representative images of sirius red staining in mice liver sections are shown. *E*, FAST and TRIGL concentration in mice serum. Data were presented as mean ± S.D., *n* ≥ 3; *, *p* < 0.05, *versus* DMSO group. *G,* suggested schematic diagram.

To further investigate whether an aged niche factor of Gal-3 pushes stemness exhaustion and therefore contributes to liver dysfunction, we used TD139, a novel inhibitor of Gal-3 (34, 35), to block the binding of Gal-3 to receptors in DEX-induced mice. We found that TD139 blocked the CD133^+^ progenitor cells expansion and biliary proliferation in liver (Fig. 7*A*). We also detected cellular signaling in mice liver, and found that TD139 treatment also recovered the AMPK phosphorylation (Fig. 7*B*). Then we tested the stemness makers using qPCR, the expressions of p16, which was responsible for quiescence maintenance, and CD133, SOX9, CK19, myc, and HNF4A were remarkably restored after TD139 treatment (Fig. 7*C*). Therefore, blocking Gal-3 pushed progenitor cells keeping in a quiescent state and protected its stemness. We also conformed the effect of TD139 treatment on liver function. Weight gain and excess fat around the abdomen is a common effect of high levels of cortisol. We found that lipid accumulation (Fig. S1B) and fibrosis were relieved (Fig. 7*D*), the AST (aspartate aminotransferase) level was decreased, and liver synthetic function was restored (Fig. 7, *E* and *F*). Together, TD139 restores progenitor cells in a quiescent state and keeps liver function well. Thus, our study suggests the DEX-induced aged niche triggers proliferation through a Gal-3/CD133/AMPK/p16 axis (Fig. 7*G*).

## Discussion

Our data demonstrate that elevated levels of Gal-3 signaling directed from aged niche leads to the loss of hepatic progenitor cells quiescence, which diminishes stemness and liver function in the long-term. In support of our data, aged hepatic progenitor cells are more active and proliferative in the aged niche induced by GC stress. It is possible that a consequence of aging across stem cell niches is their inability to retain stem cells in a quiescent state.

Retention of quiescence is essential for maintenance of stem cell function (18, 24). Quiescence of adult stem cells is regulated at multiple levels. The environment plays a significant role in stem cell proliferation during repair. We now show that aged hepatic progenitor cell niche under GC stress becomes stimulatory, driving stem cells of quiescence, suggesting that the niche is dominant during GC-induced damage. We demonstrate that Gal-3 links the aged niche and progenitor cells. Gal-3 acts as a ligand of stem cell marker CD133 in a glycosylation dependent manner and subsequently causes signaling change. In the DEX-induced model, Gal-3 partly derives from senescent hepatocytes under GC stress, hepatocytes induced by DEX show high levels of senescent-associated β-gal (Fig. 4*B*). In fact, SASP consisting of inflammatory cytokines, growth factors, and proteases is another characteristic feature of senescent cells (36). However, the elevated Gal-3 might be derived from other cell types, such as macrophages (37), as we find elevated levels of cytokinesin in the DEX model (Fig. 3*A*) and co-localization of Gal-3 and macrophage marker (Fig. 4*D*). Gal-3 is a member of the galactoside-binding lectins, either intra- or extracellularly, initiating inflammatory and fibrotic activity (38, 39). The activation of macrophages and recruitment and activation of myofibroblasts, two key phenomena in fibrosis, are both dependent on Gal-3. It is possible that Gal-3 and other cytokines regulate activation of macrophages, then the Gal-3 derived from activated macrophages together with senescent hepatocytes cause progenitor cell proliferation. In addition, TD139 has been shown to be a potent inhibitor of the galactoside-binding pockets of galectins (34, 35). We also find TD139 also inhibits the protein level in mice liver, suggesting that TD139 may also have an effect on the populations of cells expressing Gal-3. Together, elevated levels of Gal-3 signaling disrupt the stem cell quiescence.

Hepatic stem cells play a significant role on liver regeneration after damage. Hybrid hepatocytes (HybHP) are a subpopulation of periportal hepatocytes that are present in the healthy liver and undergo extensive proliferation and replenish liver mass after chronic hepatocyte-depleting injuries (40). Cholangiocytes act as facultative liver stem cells during impaired hepatocyte regeneration. Severe liver injuries can induce biliary epithelial cells to significantly contribute to hepatocyte regeneration through direct lineage conversion (41). The state of these hepatic progenitor cells depends on the damaged niche factors. That aged progenitor cells break quiescence under GC stress conditions was surprising considering their proliferative disadvantage in liver regenerative contexts. In support of our data, Gal-3 derived from niche instructs and activates the hepatic progenitor cells. In most tissues, there is an age-related decline in stem cell functionality but not a depletion of stem cells. We demonstrate that Gal-3 derived from aged niche triggers hepatic progenitor cell proliferation and stemness exhaustion in the long-term. Stemness loss decides the progenitor cell function. TD139 blocks Gal-3 binding to stem cell maker CD133, then maintains the stemness.

To our knowledge, this is the first identification of a ligand that specifically increase within a GC stress-induced aged niche that can promote breaks in quiescence leading to declines in stemness. Importantly, blocking the binding of Gal-3 by TD139 restores the stemness and liver function. Our results demonstrate a mechanism of DEX-induced aged niche-derived Gal-3 causes hepatic progenitor cells activation and provide a clue to drug intervention in stress-induced liver damage.

## Materials and methods

### Animals

Eight-week–old male C57BL/6 mice were purchased from Shanghai Model Organisms Center, Inc. (Shanghai, China). The standard chow and water were provided *ad libitum* and all mice were housed under the controlled room temperature (23 ± 3 °C) and relative humidity (70 ± 10%) on a 12-h light/12-h dark cycle. For the GC stress-induced model, C57BL/6 mice were treated with either Dex or PBS (control) via intraperitoneal injections at a dose of 25 mg/kg per day for 10 days. For TD139 administration, addition of TD139 intraperitoneal injections at a dose of 15 mg/kg per day were used during the last 5 days of Dex treatment. All mice were killed after 10 days. They were sacrificed later after operation. Blood samples were collected for biochemistry analysis (Roche, cobas c 311). Part of the liver was fixed in 4% paraformaldehyde for paraffin blocks, and the remaining fresh liver tissues were frozen by liquid nitrogen and stored at −80°C for Western blotting or qRT-PCR analysis. The experimental protocol was reviewed and approved by the ethical committees of the School of Life Science, Fudan University.

### Histology, ORO, sirius red staining

Paraffin-embedded tissue sections were routinely stained with H&E using standard protocols. Staining with sirius red was performed for visualizing liver tissue fibrosis. For sirius red staining, sections were stained for 90 min with Direct Red 80 dye (Sigma-Aldrich). All images were captured using a Zeiss microscope. The percentage of liver tissue area occupied by fatty hepatocytes or the sirius red-positive area was obtained from six different fields in each sample. The average values were considered as hepatic steatosis index and fibrosis index.

### SA-β-Gal staining

Senescence β-galactosidase staining kit was purchased from Cell Signaling Technology (catalog number 9860). Cytochemical staining of SA-β-Gal activity was performed on formaldehyde-fixed cultured cells following procedures. After staining, the number of SA-β-Gal–positive and –negative cells were counted under a bright-field microscope in six randomly selected fields.

### Western blotting

Cells were lysed with buffer containing 0.5% SDS, 5% mercaptoethanol, and 1% protease inhibitor mixture. Protein concentration was determined using a BCA protein assays kit (23225, Thermo Scientific). Proteins were subjected to SDS-PAGE and then transferred to a polyvinylidene fluoride membrane. After blocking with 5% BSA for 2 h at room temperature, the membranes were, respectively, incubated with the indicated antibodies overnight at 4 °C. The dilution ratios of primary antibodies are shown in Table S1. After incubation with horseradish peroxidase-conjugated secondary antibodies for 2 h at room temperature, protein expression was detected by the enhanced chemiluminescent method and imaged with a Molecular Imager System (Bio-Rad, USA).

### Analysis of mRNA expression

Total RNA was reverse transcribed using TRIzol reagent (Invitrogen, USA) according to the manufacturer's instructions. The RNA was reverse transcribed to cDNA with Takara SYBR Premix Ex Taq^TM^ and subsequently underwent quantitative real-time PCR utilizing a StepOnePlus Real-time PCR Instrument (Applied Biosystems, USA). Primer sequences used in qRT-PCR are listed in Table S2. Genes were normalized to β-actin. Relative mRNA expression was calculated using the comparative cycle method (2^−^Δ^Δ^*^Ct^*).

### ELISA

To quantify the amount of Gal-3 within mice serum, a mouse galectin-3 LGALS3 ELISA kit (EMLGALS3, Thermo) was performed according to the manufacturer´s instructions. Using a microplate reader set to 450 and 550 nm to determine the optical density (OD) of each well and then construct a standard curve to calculate the corresponding concentrations.

### Cell culture

CD133^+^ cells were cultured in the DMEM/F-12 media supplemented with B27 lacking vitamin A, 2 μg/ml of heparin (Sigma), 20 ng/ml of epidermal growth factor (Chemicon), and 10 ng/ml of FGF-2 (Chemicon). HEK293T cells and L02 cells were maintained in DMEM (GIBCO BRL) with 10% FBS (GIBCO BRL), 100 units/ml of penicillin, and 50 mg/ml of streptomycin (GIBCO BRL) at 37 °C in a humidified 5% CO_2_ incubator. For serum starvation treatment of L02 cells, cells were washed four times with PBS and cultured in DMEM without FBS for 48 h.

### Transient transfection with siRNAs

Cells seeded at 5 × 10^3^ cells per well in a 6-well–dish were grown overnight. The cells were then transiently transfected with 100 nm (each) specific siRNAs by use of Lipofectamine RNAi MAX transfection reagent (Invitrogen) according to the manufacturers' instructions. siRNAs were designed to target the following specific mRNA sequences: human p16 siRNA1, 5'-CCAACGCACCGAAUAGUUA-3'; human p16 siRNA3, 5'-CCGCAUAGAUGCCGCGGAA-3'; human lgals3 siRNA3, 5'-GCAAUACAAAGCUGGAUAA-3'; human CD133 siRNA, 5'-GCUCAGAACUUCAUCACAA-3'. A nonspecific (ns) siRNA (5'-UUCUCCGAACGUGUCACGU-3') was used as a negative control.

### Isolation of CD133^+^ Huh7 cells

CD133^+^ cells were isolated from hepatoma cell lines as previously described (42). CD133^+^ and CD133^−^ cells were separated through magnetic cell sorting with a CD133 Cell Isolation Kit (MiltenyiBiotec).

### CD133 protein purification and GST pulldown assay

To purify the CD133 protein, HEK293T cells transfected with CD133 plasmid were lysed at 4 °C for 2 h by use of lysis buffer (150 mm NaCl, 100 mm Tris (pH 8.0), 0.5% Triton X-100, 1 mm EDTA, protease inhibitor mixture, 1 mm phenylmethylsulfonyl fluoride), and then insoluble materials were removed by centrifugation at 12,000 × *g* for 10 min. The supernatants of cell lysates were precleared by incubation with protein G-agarose (Roche) at 4 °C for 2 h. Anti-FLAG antibody-conjugated agarose gel (FLAG-M2; Sigma) was incubated with the cell lysates overnight under constant agitation at 4 °C. After incubation, the beads with antibody were washed three times in lysis buffer to remove nonspecific binding proteins from the agarose. FLAG peptide (100 g/ml) was used to elute FLAG-tagged CD133. The eluate was concentrated to a volume of 20 μl by use of an ultrafiltration tube (Millipore). The purity of enriched CD133 protein was determined by Coomassie Blue staining.

For GST pulldown assay, the CD133 protein and GST-Gal-3 protein (Proteintech) were supplemented in the lysis buffer containing GSH high capacity magnetic-agarose beads (Sigma), according to manufacturer' s instructions. The protein complexes were then analyzed by Western blotting.

### EdU staining

Once cells reached 50% confluence, they were treated with 10 μm EdU for 2 h at 37 °C. Subsequent fixation and staining were performed using the kFluor488-EdU assay kit (Keygen Biotech) according to the manufacturer's protocol.

### Cell proliferation assays

Cell proliferation assays were analyzed using the commercial Cell Counting Kit (CCK8) in accordance with the manufacturer's instructions. In brief, cells were seeded onto 96-well–plates (Corning) at a density of 2 × 10^3^ cells/well, and incubated for 2 h to allow cell adherence to the plate. CCK8 reagents (Dojindo, CK04) were added to each well and incubated for 2 h at 37 °C. Absorbance at 450 nm was measured using Microplate Reader (Bio-Tech Instruments, USA). The results were plotted as mean ± S.D. of three separate experiments.

### Extracellular protein preparation

Conditioned cell medium was subjected to differential centrifugation at 4 °C as follows: 300 × *g* for 5 min, 500 × *g* for 5 min, 1,200 × *g* for 20 min, and 10,000 × *g* for 30 min. The 10,000 × *g* supernatant was subjected to filtration with 0.22-μm low protein-binding Millex-GV filters (Millipore); the filtrate was then concentrated by use of Amicon Ultracel-10K tubes (Millipore) according to the manufacturer's instructions. The concentrate was diluted 1:1 (v/v) with PBS and then concentrated by use of Amicon Ultracel-10K tubes (Millipore). The resulting concentrate was resuspended in Laemmli buffer and analyzed by Western blotting.

### Immunofluorescence assay

Cells were grown on coverslips, fixed with 4% paraformaldehyde for 40 min at room temperature, and gently washed three times with PBS. Liver tissue slides were fixed using the same processes. Slides were then blocked with PBS containing 5% normal serum and 0.3% Triton X-100. Cells or tissues were sequentially incubated overnight at 4 °C with primary antibodies. After washing three times with PBS, cells were incubated with anti-rabbit/mouse antibody–Alexa Fluor 488 (1:400) and anti-mouse/rabbit antibody–Alexa Fluor 594 (1:500) for double immunofluorescence staining. Nuclei were counterstained with Hoechst 33258 (10 g/ml). Immunofluorescence images were collected on a Leica TCS SP8 confocal microscope.

### Statistical analyses

Statistical analysis of the data were calculated by using two-tailed Student's t tests (*, *p* < 0.05; **, *p* < 0.01) on GraphPad Prism. All values included in figures represent mean ± S.D. *Error bars* represent S.D. Data are representative of at least three independent experiments.

## Data availability

All data described are located within the manuscript.
