# Supplementary material for: Secretory galectin-3 induced by glucocorticoid stress triggers stemness exhaustion of hepatic progenitor cells
Source: J Biol Chem. 2021 Jan 13;295(49):16852–62. doi: 10.1074/jbc.RA120.012974 (PMC7864077; doi:10.1074/jbc.RA120.012974)
Supplement: Supplementary file 1 [file mmc1.docx]

**Supporting information**

**1. Figure Sup**

**
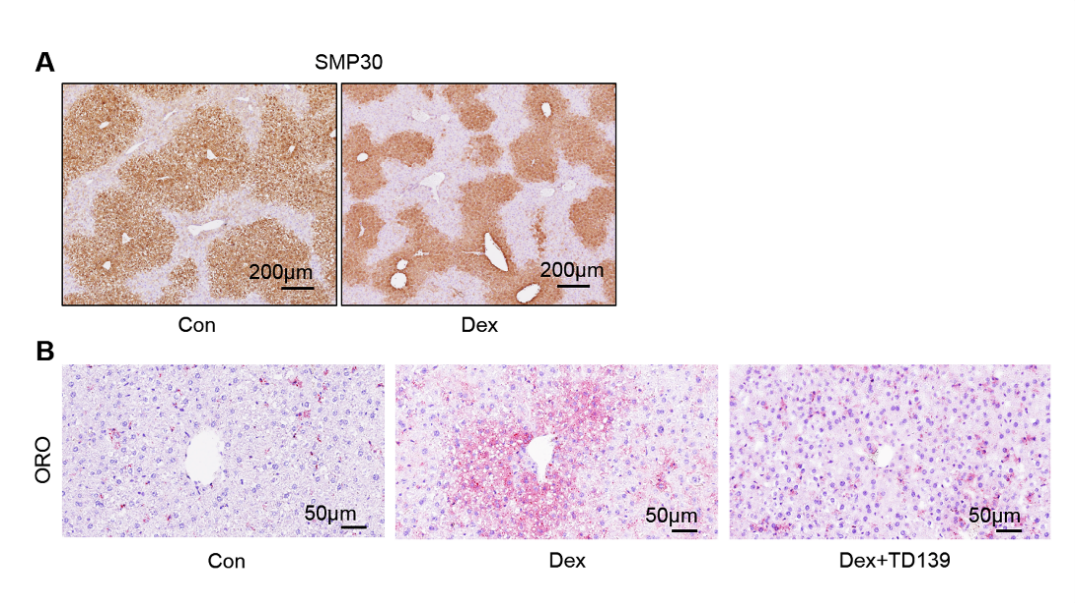
**

Figure Sup. A IHC analysis of SMP30 for mice liver (n=3), scale bar was shown as indicated.

B Representative pictures of Sirius red staining in mice liver sections were shown. Scale bar, 50 μm.

**2. Table S1 antibodies and use.**

| **Antibodies** | **Company** | **Catalog No.** | **Dilution** | **Use** |
| --- | --- | --- | --- | --- |
| smp30 | abcam | ab233007 | 1:200 | IHC |
| smp30 | abcam | ab233007 | 1:1000 | Western Blot |
| CD133(W6B3C1) | Miltenyi Biotec | 130-092-395 | 1:200 | Western Blot |
| CD133(W6B3C1) | Miltenyi Biotec | 130-092-395 | 1:40 | Immunofluorescence |
| CD133-R | abcam | ab19898 | 1:1000 | Western Blot |
| CD133-R | abcam | ab19898 | 1:200 | IHC |
| CD133-R | abcam | ab19898 | 1:100 | Immunofluorescence |
| SOX9 | abcam | ab185966 | 1:1000 | Western Blot |
| SOX9 | abcam | ab185966 | 1:1000 | IHC |
| CK19 | Cell Signaling Technology | 12434s | 1:600 | IHC |
| CK19 | Cell Signaling Technology | 12434s | 1:1000 | Western Blot |
| β-actin-HRP | Proteinthch | HRP-60008 | 1:2000 | Western Blot |
| gal-3-R | abcam | ab76245 | 1:2000 | Western Blot |
| gal-3-R | abcam | ab76245 | 1:50 | IHC |
| gal-3-M | abcam | ab2785 | 1:1000 | Western Blot |
| gal-3-M | abcam | ab2785 | 1:200 | Immunofluorescence |
| gal-3-R | abcam | ab31707 | 1:2000 | Western Blot |
| HMGB1 | Cell Signaling Technology | 6893T | 1:1000 | Western Blot |
| HSP90 | Santa Cruz Biotechnology | sc-13119 | 1:400 | Western Blot |
| C-MYC | Cell Signaling Technology | 5605T | 1:1000 | Western Blot |
| ALIX | abcam | ab186429 | 1:1000 | Western Blot |
| GST (91G1) Rabbit mAb | Cell Signaling Technology | 2625T | 1:1000 | Western Blot |
| GST (91G1) Rabbit mAb | Cell Signaling Technology | 2625T | 1:100 | Immunofluorescence |
| P-FAK | abcam | ab81298 | 1:1000 | Western Blot |
| FAK | abcam | ab40794 | 1:1000 | Western Blot |
| P-AMPK | Cell Signaling Technology | 2535T | 1:1000 | Western Blot |
| AMPK | Cell Signaling Technology | 5831T | 1:1000 | Western Blot |
| p16 | abcam | ab54210 | 1:200 | IHC |
| p16 | abcam | ab211542 | 1:1000 | Western Blot - tussue |
| P16 | Cell Signaling Technology | 92803T | 1:1000 | Western Blot-cell |
| P21 | Cell Signaling Technology | 2947T | 1:1000 | Western Blot |
| P27 | Cell Signaling Technology | 3698S | 1:1000 | Western Blot |
| PCNA | Cell Signaling Technology | 2586T | 1:10000 | IHC |
| hnf4a | abcam | ab199431 | 1:400 | IHC |
| F4/80 | Cell Signaling Technology | 30325s | 1:200 | Immunofluorescence |
| anti-mouse IgG/Alexa Fluor 488 | Cell Signaling Technology | 4408 | 1:400 | Immunofluorescence |
| anti-rabbit IgG/Alexa Flour 594 | Cell Signaling Technology | 8889 | 1:500 | Immunofluorescence |
| Goat anti-rabbit IgG/Alexa Fluor 488 | Thermo Fishser | A11008 | 1:400 | Immunofluorescence |
| Goat anti-mouse IgG/Alexa Flour 594 | Thermo Fishser | A27016 | 1:500 | Immunofluorescence |

**3.Table S2 Primers**

| Gene symbol | Forword 5'-3 | Reverse 5'-3' |
| --- | --- | --- |
| Mouse p16 | GAACTCTTTCGGTCGTACCC | CGAATCTGCACCGTAGTTGA |
| Mouse p53 | CTCTCCCCCGCAAAAGAAAAA | CGGAACATCTCGAAGCGTTTA |
| Mouse p21 | CCTGGTGATGTCCGACCTG | CCATGAGCGCATCGCAATC |
| Mouse p27 | TCAAACGTGAGAGTGTCTAACG | CCGGGCCGAAGAGATTTCTG |
| Mouse IFN-γ | ATGAACGCTACACACTGCATC | CCATCCTTTTGCCAGTTCCTC |
| Mouse IFN-β | TCCGAGCAGAGATCTTCAGGAA | TGCAACCACCACTCATTCTGAG |
| Mouse IL-6 | TAGTCCTTCCTACCCCAATTTCC | TTGGTCCTTAGCCACTCCTTC |
| Mouse CXCL10 | GCCGTCATTTTCTGCCTCA | CGTCCTTGCGAGAGGGATC |
| Mouse LGALS3 | AGACAGCTTTTCGCTTAACGA | GGGTAGGCACTAGGAGGAGC |
| Mouse TNFα | CAGGCGGTGCCTATGTCTC | CGATCACCCCGAAGTTCAGTAG |
| Mouse MMP3 | ACATGGAGACTTTGTCCCTTTTG | TTGGCTGAGTGGTAGAGTCCC |
| Mouse β-Actin | GGCTGTATTCCCCTCCATCG | CCAGTTGGTAACAATGCCATGT |
| Mouse CD133 | ACTGGGGCTGTGTGGAAAG | GCATTGAAGGTATCTTGGGTCTC |
| Mouse CD34 | AAGGCTGGGTGAAGACCCTTA | TGAATGGCCGTTTCTGGAAGT |
| Mouse sox9 | AGTACCCGCATCTGCACAAC | ACGAAGGGTCTCTTCTCGCT |
| Mouse CK19 | GTTCAGTACGCATTGGGTCAG | GAGGACGAGGTCACGAAGC |
| Mouse ATXN1 | CTCCCAAGAAACGTGAGATCC | CCATTCCTTGTAAACCATGCTCC |
| Mouse myc | ATGCCCCTCAACGTGAACTTC | GTCGCAGATGAAATAGGGCTG |
| Mouse nanog | TCTTCCTGGTCCCCACAGTTT | GCAAGAATAGTTCTCGGGATGAA |
| Mouse HNF4a | CACGCGGAGGTCAAGCTAC | CCCAGAGATGGGAGAGGTGAT |
| Human β-Actin | GGACTTCGAGCAAGAGATGG | AGGAAGGAAGGCTGGAAGAG |
| Human p16 | GGGTTTTCGTGGTTCACATCC | CTAGACGCTGGCTCCTCAGTA |
| Human CD133 | TGGATGCAGAACTTGACAACGT | ATACCTGCTACGACAGTCGTGGT |
| Human CD34 | CTACAACACCTAGTACCCTTGGA | GGTGAACACTGTGCTGATTACA |
